# Special Issue: “Gastrointestinal Cancers and Personalized Medicine”

**DOI:** 10.3390/jpm12030338

**Published:** 2022-02-24

**Authors:** Stefania Nobili, Enrico Mini

**Affiliations:** 1 Department of Neurosciences, Imaging and Clinical Sciences, “G. d’Annunzio” University of Chieti-Pescara, 66100 Chieti, Italy; 2 Center for Advanced Studies and Technology (CAST), “G. d’Annunzio” University of Chieti-Pescara, 66100 Chieti, Italy; 3 Department of Health Sciences, University of Florence, 50139 Florence, Italy; enrico.mini@unifi.it; 4 DENOTHE Excellence Center, University of Florence, 50139 Florence, Italy

Gastrointestinal cancers represent more than 25% of all diagnosed cancers and more than 36% of cancer-related deaths worldwide [1]. Unfortunately, screening strategies are still limited. They are, in fact, available only for colorectal, gastric and esophageal cancers [2]. However, despite these early diagnostic opportunities, gastrointestinal cancers, including pancreatic, hepatobiliary, small bowel carcinomas and other uncommon cancers, such as anal canal cancer, neuroendocrine tumors of the gastrointestinal tract, primary gastric and intestinal lymphomas, and gastrointestinal stromal tumors (GISTs), are frequently diagnosed at an advanced stage, when treatment options are limited and cure is not possible. Moreover, a very high percentage of patients, about 50%, diagnosed with early potentially curable gastrointestinal cancers, will develop recurrent disease despite surgery, radiation therapy, and pharmacological treatment during the course of the disease [3]. Taken together, these conditions are responsible for poor prognosis in these tumors. Thus, despite current available knowledge on molecular determinants involved in the initiation and progression of cancer [4], there is an urgent clinical need to further improve our biological knowledge of gastrointestinal evolutionary processes toward increased dysregulation, heterogeneity, and the escape from immunosurveillance as well as from pharmacological treatment control [5,6].

Such complex processes substantially involve all types of molecules (e.g., nucleic acids, proteins, metabolites) and involve several cell types, such as transformed epithelial or mesenchymal cells, or other tumor microenvironment cells, including immune cells.

The development and availability of the newest biotechnologies that add knowledge to the field of cancer research strongly contribute to new cancer achievements aimed at discovering and validating novel molecular biomarkers predictive of prognosis and drug response (efficacy/toxicity) in gastrointestinal cancers. A number of cancer biomarkers, mainly represented by somatic alterations in tumor cells (e.g., in the *RAS*, *RAF*, *MMR*, *HER-2* and *KIT* genes), have been identified and validated as clinically useful biomarkers to predict patient prognosis and drug response in gastrointestinal cancers such as colorectal cancer, gastric cancer and GISTs, thus directly contributing to therapeutic decisions. 

However, due to the high level of tumor heterogeneity, not only among patients but also among tumor sites in the same patient, the possibility of employing effective personalized medicine for all patients still represents a relevant challenge. Currently, a plethora of potential biomarkers predictive of prognosis or drug response have been suggested [7]. Their detection in tissue and/or in bio-fluidic samples has the potential to improve clinical oncology practice.

In addition, a pharmacogenetic approach, represented by the analysis of germline polymorphisms in genes that play a main role in the ADME of anticancer drugs, has also been progressively introduced into clinical practice for the prediction of the risk of toxicity related to these drugs. However, few polymorphisms in pharmacogenes have been shown to be responsible for drug toxicity. Thus, this aspect still represents a major issue in cancer care.

This Special Issue is designed to provide information on new biomarker research in the area of gastrointestinal tumors that could be useful for innovative personalized management and precision medicine modalities for individualized care.

Gastrointestinal cancers are often diagnosed at advanced stages when therapeutic options are limited. Liquid biopsy, a non-invasive procedure, widely investigated in recent years and already applied to monitor cancer progression and drug resistance, mainly in lung cancer, could also be successfully used to diagnose cancers at early stages. Liquid biopsy, usually performed in blood serum, could be obtained by several different body fluids [8]. Among gastrointestinal cancers, pancreatic cancer could greatly benefit from this opportunity. In fact, although it is only the 12th most common cancer, it is the 6th most common cause of cancer death [1]. Thus, it would be crucial to identify a strategy able to diagnose pancreatic cancer in advance, before tumor development. The incidence of pancreatic cysts is about 2% in adults and neoplastic cysts account for 10%–15% of all pancreatic cystic lesions. Although their risk to change in malignant lesions is low, if this occurs, the patient prognosis will be very poor [9].

Hermoso-Durán et al. [10] investigated cyst liquid samples from patients, and the proteomic differences between pancreatic benign and premalignant cysts. To perform such an evaluation the authors used an approach previously used on serum or plasma, named “thermal liquid biopsy” (TLB), which they adapted to cyst liquid samples. Based on the TLB thermograms, cyst profiles were clustered according to their clinical assessment. The authors also elaborated a new TLB serum score based on the specific parameters reflecting differences between cysts. Results were encouraging although the number of analyzed samples was small. The availability of a dedicated TLB as a diagnostic tool for serum samples from patients with pancreatic cysts of which the nature is unknown could represent a relevant advantage in the diagnosis of premalignant lesions of pancreatic cancer. 

The prognosis of patients affected by colorectal cancer, one of the most incident and lethal cancers worldwide [1], is highly variable, mainly dependent on the stage at diagnosis. Through the years, many efforts have been made to identify and validate molecular biomarkers predictive of prognosis and/or drug response in this neoplasm. In recent years, interesting examples of predictors of prognosis concern the colorectal cancer molecular subtypes that have been obtained by unsupervised transcriptomic approaches, i.e., consensus colorectal cancer molecular subtypes (CMSs) [11] and colorectal cancer intrinsic subtypes (CRIS) [12]. However, the clinical utility of such classifications for the single patient has yet to be established. Potential biomarkers predictive of response to adjuvant chemotherapy in the early colorectal cancer stages (i.e., stage II-III) have been suggested, for instance from validated transcriptomic [13,14,15] or genetic [16] analyses. However, to date, biomarkers predictive of drug response represented by actionable oncogenic drivers (i.e., *RAS* wild-type and MSI-H status for anti-EGFR and anti-PD-1 monoclonal antibodies, *BRAF* V600E mutations, *NTRK* gene fusions and more recently *KRAS* G12C mutations for targeted agents) are used only in the metastatic setting [17]. 

In this framework, the review of Del Buono et al. [18] contextualized the role of the DNA mismatch repair (MMR) system in colorectal cancer precision medicine. Today, the knowledge of the MSI status provides several advantages by satisfying a number of clinical queries. In fact, MSI, due to an impaired MMR system, plays a role in the inherited predisposition to gastrointestinal cancers, and identifies a subset of colorectal cancer patients who show a substantial better prognosis and who do not obtain an advantage from adjuvant chemotherapy (i.e., low-risk stage II patients). More recently, MSI has become a key biomarker for the treatment of several tumors, including colorectal cancer, with immune checkpoint inhibitors. Thus, the evaluation of MMR/MSI is becoming part of standard care in colorectal cancer, as recommended by major oncological international societies [17].

Overall, therapeutic options in colorectal cancer are related to the cancer stage and, as mentioned above, differ from metastatic and nonmetastatic settings. Stage I and low-risk stage II patients are treated with surgery alone. High-risk stage II, stage III and stage IV (oligometastatic disease) patients are instead treated with pharmacological therapy in addition to surgery, with positive results. However, neoplastic progression due to additional dysregulated molecular events occurs in a substantial percentage of patients, limiting the efficacy of the available drugs administered as adjuvant or neoadjuvant therapies. This occurrence stimulates the search for biomarkers able to predict colorectal cancer prognosis in order to plan preventative pharmacological strategies for patients at high risk of disease progression as well as biomarkers predictive of drug response, in order to avoid the administration of inactive drugs to resistant patients. Immunoscore is a further example of tumor biomarker able to predict disease prognosis in early-stage colorectal cancer [19]. Instead, tumor mutational burden is not yet a recommended biomarker for the prediction of pembrolizumab efficacy in colorectal cancer due to the limited data available in this patient population [17].

The study of Rhyner Agocs et al. [20] evaluated the predictive role of the expression of the lymphocyte-activation gene 3 (LAG-3) in the outcome of 143 stage II colon cancer. LAG-3 is an inhibitory immune-related molecule mainly expressed on T cells, but also on B cells and dendritic cells. LAG-3 may synergize with the PD-1/PD-L1 pathway and is closely related to CD4. The upregulation of LAG-3 on immune cells downregulates T cell expansion and cytokine secretion, and thus contributes to an immunosuppressive microenvironment. In particular, the presence of LAG-3 was evaluated by immunohistochemistry in formalin-fixed paraffin-embedded (FFPE) tissues on tumor-infiltrating lymphocytes (TILs) in the tumor center and tumor front to assess its impact on the survival of stage II colon cancer patients. The authors found no correlations between LAG-3 expression and clinical/pathological characteristics, although they observed a higher percentage of MMR-deficient colon cancers when LAG-3-positive TILS were present. In relation to the primary study end-point, i.e., disease-free survival, the authors found a significant association between the presence of LAG-3 in the tumor front and prolonged disease-free survival. This significant correlation was maintained even when only MMR-proficient colon cancer, (i.e., the majority of the analyzed tumors), were considered. Moreover, in this case, such a correlation was limited to TILs localized at the tumor front. Thus, this manuscript identified LAG-3 as a biomarker potentially useful in predicting patient prognosis in stage II colon cancer, including MMR-proficient tumors.

Similarly, Peyravian et al. [21] analyzed a panel of candidate genes (i.e., 20 genes) whose expression was potentially involved in the development of lymph node metastases in 100 colorectal cancer patients. The selected genes were chosen according to their role in key cancer processes such as carcinogenesis, tumor growth, tumor invasion and metastasis. Overall, about 60% of patients initially diagnosed as stage I-III, were lymph nodes negative. Hierarchical clustering analysis showed that *VANGL1*, *PCSK7*, and *ANXA3* genes were the most expressed among the study genes at mRNA level in the majority of colorectal cancer samples. However, only *VANGL1* was shown to significantly vary between lymph node-negative and -positive patients. The mRNA expression levels of *VANGL1* were also confirmed at protein level. The study also provided associations between two other study genes, *NOTCH1* and *ILR2B,* and overall survival. In particular, the high expression of *NOTCH1* and the low expression of *ILR2B* were associated with prolonged overall survival.

In metastatic colorectal cancer, Taghizadeh et al. [22] provided a molecular profile of a real-world cohort of drug refractory patients for whom no further standard treatment option was available. The molecular profile was performed by a precision medicine platform developed at the author’s institution, i.e., the Comprehensive Cancer Centre of the Medical University of Vienna. Based on the biomolecular characteristics of tumors, this study was aimed at providing information on potential further options of targeted therapy. Overall, by exploiting next-generation sequencing panels of mutation hotspots, microsatellite instability testing, and immunohistochemistry, 60 metastatic colorectal cancer samples were characterized. The analysis revealed 166 mutations in 53 patients, the five most frequent being *TP53*, *KRAS*, *APC*, *PIK3CA*, and *PTEN*. All patients had previously received cytotoxic chemotherapy combined with anti-EGFR or anti-VEGF(R) monoclonal antibodies. The study showed that, in 47% of patients, a molecularly targeted therapy could be recommended whereas the remaining were not suitable for targeted therapy due to the lack of actionable molecular targets. Overall, 20% of the study patients underwent the recommended targeted therapy. In particular, pembrolizumab was offered to four MSI-H patients, consequently obtaining control of disease in all patients and objective response in 75%. Stable disease was observed in two further patients treated with everolimus combined with raltitrexed, and with trastuzumab combined with lapatinib, respectively, according to their specific immunohistochemical and mutational characteristics (i.e., strong m-TOR expression associated with the loss of PTEN and HER2+ overexpression, respectively). Overall, this study highlights how at least a portion of heavily pretreated patients without further standard treatment options may benefit from a molecular-based treatment approach. 

Interestingly, by a rationale based on the role that the immune response and inflammation play in tumor growth and in the metastatic process, Fülöp et al. [23] evaluated the prognostic impact of the neutrophil-to-lymphocyte and lymphocyte-to-monocyte ratios (i.e., NLR and LMR) in over 1000 rectal cancer patients. The overall survival was significantly associated with increased NLR and decreased LMR, and no relationship was found between the study ratios and tumor stage, thus potentially suggesting that these markers are independent from cancer stage, even if this occurrence is controversial [24]. Moreover, NLR and LMR were also found to predict response to the neoadjuvant chemoradiotherapy to which patients underwent. In particular, the identification of a cut-off for NLR value (i.e., ≥3.11) allowed the authors to discriminate between chemoradiotherapy responsive and non-responsive rectal cancer patients, although the responsive ones had a low chance of sphincter preservation, or to obtain a complete total mesorectal excision. Although the study ratios may also be affected by factors independent of the neoplastic disease, these data warrant attention due to the high number of patients included in this analysis and deserve further investigation.

Oxaliplatin, widely used in the treatment of gastrointestinal cancers, is a highly neurotoxic agent. Acute or chronic peripheral neuropathy develops in about 90% and 40% of patients, respectively, and the latter form may strongly affect the quality of life of patients and cancer survivors. Unfortunately, to date, no remedy or antidote is available to reverse this side effect. Thus, it would be very important to identify patients susceptible to develop peripheral neuropathy before starting the oxaliplatin treatment, even though, despite the efforts of many researchers, no predictive biomarker has yet been identified. 

The review of Velasco et al. [25] discusses the status of the art of strategies that may be implemented pre-emptively to evaluate the risk of developing neurotoxicity. In particular, neurological monitoring through the evaluation of neurophysiological signs of oxaliplatin-induced neuropathy may be performed by mechanical strategies (e.g., nerve conduction tests, electromyography). However, this procedure is not part of the common clinical practice. Less invasive blood biomarkers have also been widely investigated. Genetic biomarkers, mainly represented by single-nucleotide polymorphisms in genes encoding detoxification enzymes (e.g., proteins belonging to the glutathione detoxification system), drug transporters (e.g., ATP binding proteins), proteins involved in the mechanism of action of oxaliplatin, as well as proteins implicated in neuronal functions, have drawn attention. In addition, proteins released in blood when nerve damage occurs (e.g., the protein neurofilament light chain (NfL) and nerve growth factor (NGF)) have also been suggested as predictive biomarkers of neurotoxicity. Neuroimaging strategies have also been studied as potential tools for the early detection of neurotoxicity onset.

Overall, the manuscripts included in this Special Issue highlight the need to identify and validate molecular biomarkers predictive of prognosis and drug response in gastrointestinal cancers. To satisfy this goal, biomarkers identified in retrospective studies will need to be validated in large-scale prospective clinical trials. Moreover, the availability of new and highly predictive biomarkers implies that the discovery of new anticancer drugs, specifically inhibiting these targets, can be accomplished to effectively treat patients who are potentially unresponsive to standard therapies.
